# Cutaneous Adverse Drug Reactions in Dogs Treated with Antiepileptic Drugs

**DOI:** 10.3389/fvets.2016.00027

**Published:** 2016-04-14

**Authors:** Tina Koch, Ralf S. Mueller, Britta Dobenecker, Andrea Fischer

**Affiliations:** ^1^Clinic of Small Animal Medicine, Centre for Clinical Veterinary Medicine, Faculty of Veterinary Medicine, Ludwig Maximilian University Munich, Munich, Germany; ^2^Department of Veterinary Sciences, Ludwig Maximilian University Munich, Munich, Germany

**Keywords:** epilepsy, phenobarbital, antiepileptic drug, adverse reaction, dermatologic, skin, side effects, patch test

## Abstract

Epilepsy is one of the most common neurologic disorders in dogs and life-long treatment with antiepileptic drugs (AED) is frequently required. Adverse events of AED targeting the skin are only rarely reported in veterinary medicine and the true incidence and spectrum of cutaneous reactions in epileptic dogs remains unknown. In this study, we hypothesized that cutaneous reactions commonly occur in epileptic dogs and are related to AED treatment. A retrospective case review of 185 dogs treated for epilepsy identified 20.0% with simultaneous appearance of dermatologic signs. In a subsequent prospective case investigation (*n* = 137), we identified newly appearing or distinct worsening of skin lesions following initiation of AED therapy in 10.9% of dogs treated for epilepsy (95% CI 6.8–17.7%). Cutaneous lesions were classified as probably drug-induced in 40.0% of these cases. Patch testing and intradermal testing were further investigated as potential diagnostic methods to confirm AED hypersensitivity. They were of high specificity but sensitivity and positive predictive value appeared inappropriate to recommend their routine use in clinical practice.

## Introduction

Anticonvulsant agents are well known to cause adverse drug reactions (ADR) in dogs and cats, and ADRs are a major concern for owners of epileptic dogs (1, 2). However, in those species, effects, which are not immunologically mediated such as polyphagia, polydipsia/polyuria, sedation, and ataxia, or an activation of liver enzymes predominate (3, 4). There are only few case reports in the scientific literature regarding immune-mediated hypersensitivity reactions against antiepileptic treatment in animals including blood cell dyscrasias (5, 6), idiosyncratic hepatopathy (caused by zonisamide) (7, 8), or cutaneous disorders. Superficial necrolytic dermatitis (SND) or more appropriate metabolic epidermal necrosis (MEN) was described to appear after several months to years of phenobarbital (PB) therapy and evidence supported its origin as a hepatocutaneous syndrome (9, 10). Dermatologic signs have also been a concern in dogs treated with potassium bromide (panniculitis) (11, 12) and zonisamide (erythema multiforme) (13).

Adverse drug reactions are divided into two categories: type 1 ADRs are not immune mediated and attributed to physical or chemical properties of the agent or its metabolites. They are dose dependent and predictable. Type 2 ADRs are immune mediated, idiosyncratic, and occur unpredictably. The mechanism of this type of ADR is not completely understood (14, 15). It is presumed that all four main types of immune reaction – as defined by Gell and Coombs – play a significant role in drug allergy (14). Manifestations of drug-induced allergic reactions can affect numerous organ systems and lead to liver damage, lymphadenopathy, pneumonia, or hematologic abnormalities, although cutaneous manifestations are most common (16).

In human medicine, antiepileptic drugs (AEDs) are well known to cause cutaneous ADRs in about 3% of patients treated with an AED (17). These immune-mediated hypersensitivity reactions can range from more frequent mild urticarioid or maculopapular eruptions to severe systemic reactions such as Anticonvulsant Hypersensitivity Syndrome (AHS) (17–19). It is suggested that AHS is triggered by a delayed type immune reaction (Type 4), since drug-specific T-cells appear to play a significant role (20), but several other approaches are discussed to be involved in pathogenesis such as cohesive viral infections with human herpes virus Types 6 and 7 or cytomegalovirus (21, 22). Furthermore, recent investigations indicate that genetic factors predispose to the development of an adverse reaction against AED (23), such as a genetically caused deficiency of detoxifying enzymes, which leads to an accumulation of toxic metabolites (19, 21, 22, 24). Management of ADRs in patients with epilepsy creates specific challenges as any change in AED treatment schedule may increase the risk for seizure recurrence, cluster seizures, or status epilepticus. Patch testing (PT) and intradermal testing (IDT) have been introduced in human medical practice during the last decades in order to predict the probability of an ADR against AED (20, 21).

We hypothesized that cutaneous signs occur more commonly in dogs treated with AEDs than reported in scientific literature. So, the aim of this study was to evaluate the proportion of AED-treated dogs that developed dermatologic signs and whether these signs are consistent with an ADR. A secondary goal was the investigation of PT and IDT as diagnostic methods.

## Materials and Methods

### Retrospective Evaluation of Adverse Events

Medical records of epileptic dogs seen between 01/2007 and 12/2012 were reviewed for documentation of adverse events after initiation of AEDs. Inclusion criteria were a clinical diagnosis of epilepsy (idiopathic or structural) according to published criteria (25), medical treatment with one or more AEDs, and at least one follow-up visit in the clinic at least 2 weeks after drug initiation. Exclusion criteria were presence of or strong suspicion for a systemic disease prior to initiation of AED therapy or simultaneous treatment with other drugs at the time of occurrence of adverse events. In total, 185 dogs were included. All data collected, including blood parameters (hematology, chemistry, liver function test), results of the clinical examination, as well as owner-reported adverse events, were reviewed. The records were particularly screened for appearance of adverse events after initiation of AED therapy such as polydipsia/polyuria, polyphagia, ataxia, sedation, gastrointestinal signs, pancreatitis, hepatopathy, hematologic changes, behavioral changes, respiratory signs, and in particular dermatologic abnormalities. Dermatologic signs were documented by clinicians as part of structurally conducted history and examination. Pancreatitis was assumed if specific canine pancreatic lipase was increased and additionally matching clinical signs and/or matching ultrasonographic findings were present. Hepatopathy was assumed if blood values (decreased albumin, decreased total protein, increased bilirubin, increased ammonia, and increased bile acids in liver function test) and clinical signs indicated it, confirmed by ultrasonographic findings. Each adverse event that occurred after AED initiation was noted, regardless of how long after drug initiation it occurred or whether it resolved again during therapy. Furthermore, we attempted follow-up calls to the owners of the dogs with noted dermatologic signs in order to obtain further information about the causality between occurrence of dermatologic signs and AED therapy.

Then, each case of registered cutaneous signs was classified as a doubtful, possible, probable, or definite ADR, as defined by Naranjo et al. (26) (Table 1). The probability category was assigned from the total score as followed: <0/0 = doubtful, 1–4 = possible, 5–8 = probable, 9/>9 = definite (26).

**Table 1:** **Classification of skin lesions with the Naranjo probability index**.

Question	Yes	No	Do not know
Are there previous conclusive reports on this reaction?	+1	−1	0
Did the adverse event appear after the suspected drug was administered?	+2	−1	0
Did the adverse reaction improve when the drug was discontinued or a specific antagonist was administered?	+1	0	0
Did the adverse reaction reappear when the drug was readministered?	+2	−1	0
Are there alternative causes (other than the drug) that could on their own have caused the reaction?	−1	+2	0
Did the reaction reappear when a placebo was given?	−1	+1	0
Was the drug detected in the blood (or other fluids) in concentrations known to be toxic?	+1	0	0
Was the reaction more severe when the dose was increased or less severe when the dose was decreased?	+1	0	0
Did the patient have a similar reaction to the same or similar drugs in any previous exposure?	+1	0	0
Was the adverse event confirmed by any objective evidence?	+1	0	0

*Assignment of probability scores: total score ≤0 = doubtful, 1–4 = possible, 5–8 = probable, and ≥ 9 = definite (26)*.

### Prospective Evaluation of Cutaneous Reactions

Dogs diagnosed with epilepsy (idiopathic or structural) and treated with AEDs between 01/2013 and 12/2014 were prospectively included and monitored for any new appearance of cutaneous signs after initiation of AED. Whenever feasible, complete dermatologic examination was performed by an ECVD diplomate or resident which routinely included detailed dermatologic history, visual inspection of the lesions, skin scrapings, and cytologic examination in order to describe the appeared lesions as precisely as possible and to exclude other potential causes such as ectoparasites and infections. Inclusion and exclusion criteria were identical to the ones described for the retrospective evaluation. Owners of dogs with dermatologic signs were asked to fill in a standardized questionnaire or alternatively answer a follow-up phone call. Based on this information, cutaneous signs were ranked using the Naranjo probability index as described for the retrospective data.

### Evaluation of Patch Test and Intradermal Test

#### Dogs

Patch test (PT) and IDT were performed in six dogs with a clinical diagnosis of epilepsy, which had developed skin lesions after initiation of PB monotherapy (four dogs) or combination therapy (two dogs).

#### Control Groups

Ten laboratory Beagle dogs (two males, eight females, median age 3.8 years; range: 2–4 years) that had never received any AED and therefore were most likely not sensitized to those allergens and seven dogs with a clinical diagnosis of idiopathic epilepsy (three males, four females, three Border collies, two Labrador Retrievers, one Beagle, and one German shepherd mix; median age: 2.5 years, range: 1.5–9 years) currently treated with PB monotherapy (2) or combination therapy [two PB/potassium bromide (KBr) and three PB/levetiracetam (LEV)] without any previous or current dermatologic signs.

In the laboratory Beagle dogs, the PT included PB, potassium bromide, LEV, gabapentin, and zonisamide, and the IDT included PB and potassium bromide. The privately owned dogs were patch tested for those AEDs, which they currently received or had received previously. PB and, if the AED treatment included it, potassium bromide were additionally used in the IDT.

#### Patch Test

For the patch test, two different concentrations (5 and 10%) of petrolatum-solved agents were compounded for each AED (Table 2). Tablets were grounded with a tablet grinder to an instant powder, which was solved in petrolatum. Approximately 0.2 cc of the prepared solutions were placed in Finn chambers of 12 mm diameter. Pure petrolatum was used as negative control. The Finn chambers were placed firmly on the clipped skin on the lateral thorax and carefully fixed by surgical tape as described previously (27). The dogs wore a body suit for 48 h to prevent shifting of the chambers (27–29). After 48 h, the chambers were removed, and the skin reactions were evaluated as described previously (Table 3) (27).

**Table 2:** **Antiepileptic agents unsed in patch tests**.

Active substance	Trade name	Concentration per pill (mg)
Phenobarbital	Luminal Vet^®^	100
Potassium bromide	Libromide^®^	325
Levetiracetam	Keppra^®^	100
Gabapentin	Gabapentin, 1A Pharma^®^	100
Zonisamide	Zonegran^®^	50

**Table 3:** **Evaluation of patch test sites**.

–	No visible reaction or irritation
1+	Mild erythema
2+	Moderate erythema
3+	Severe erythema
++	Erythema and induration or edema (papules)
+++	Erythema with vesiculation or more severe reactions

#### Intradermal Test

Intradermal testing was performed with PB and potassium bromide. The corresponding amount of 200 mg effective agent was solved in 2 cc of sterile isotonic saline solution, resulting in a 10% dilution. To detect the minimum drug dilution not triggering a positive reaction when injected into the skin of a healthy, non-sensitized dog, serial dilutions were prepared (10, 1, 0.1, and 0.01%) and injected intradermally into the skin of a healthy laboratory Beagle dog, which had never received any AEDs. Positive reactions were seen with the 10 and 1% dilution. Consequently, a 0.1% dilution of both drugs was used subsequently in this study. An area of 20 cm × 15 cm on the lateral thorax was clipped, and the injection sites were marked by an indelible felt pen. Approximately 0.05 cc of each solution as well as a positive (histamine 1:100,000) and a negative control (sterile isotonic saline solution) were injected intradermally using an insulin syringe (0.33 mm × 12 mm) as previously described (30). After 15 and 25 min, the skin reaction and diameter of the wheal at the drug injection site were compared to the negative and positive control and graded either negative (−) or positive as +, ++, or +++, depending on strength of reaction.

Sensitivity, specificity, and positive and negative predictive value of PT and IDT and their 95% confidence intervals were calculated.

This animal experiment was approved by the government, under the reference 55.2-1-54-2532-4-13. The laboratory dogs were housed according to the prescribed conditions of the German animal protection law. Epileptic dogs were privately owned patients of the clinic. All animal owners signed informed consent prior to study participation.

## Results

### Retrospective Evaluation of Adverse Events

In total, 185 dogs (84.9% idiopathic epilepsy and 15.1% structural epilepsy) fulfilled the inclusion criteria. The 52.4% of the retrospective cases were treated with AED monotherapy [93 with PB, 3 with potassium bromide (KBr), and 1 with levetiracetam (LEV)] and 47.6% with a combination of AED (66 with PB/KBr, 5 with PB/LEV, 10 with PB/KBr/LEV, 3 with PB/KBr/gabepentin, 1 with PB/KBr/zonisamide, 1 with PB/zonisamide, 1 with PB/KBr/LEV/zonisamide/pregabalin, and 1 with PB/pregabalin/zonisamie/lacosamide).

Clinical signs that occurred after initiation of antiepileptic treatment and were considered adverse events are listed in Table 4. Most frequently noted were ataxia (27.1%), sedation (23.8%), and polyphagia (20.5%), while pancreatitis (5.4%) and hepatopathy (2.2%) were less frequently seen. Neutropenia occurred in six dogs (3.2%). Cutaneous signs were documented in 20.0% of the cases, 30 of them diagnosed with idiopathic epilepsy. The dermatologic signs ranged from solely pruritus/alopecia (*n* = 23), skin lesions such as papules, pustules, or erythema (*n* = 13) to severe reactions such as epidermal necrosis (*n* = 1). In total, 26 owners were available for a detailed follow-up phone interview. These owner interrogations revealed that four dogs already had dermatologic signs before AED initiation and two owners could not surely remember whether the dermatologic signs occurred before or after AED therapy. Based on these information and on the clinical records, the Naranjo index (26) was used in order to classify the dermatologic signs as probably related to AED treatment in 2 dogs (5.4%), possible in 22 dogs (59.5%), and doubtful in 13 dogs (35.1%).

**Table 4:** **Adverse events during AED therapy (*n* = 185)**.

	Polyuria/polydypsia	Polyphagia	Sedation	Ataxia	Gastro-intestinal disorders	Pancreatitis	Changes in behavior	Respiratory signs	Dermatol. signs	Hepatopathy
PB (*n* = 93)	15	19	16	15	4	3	5	0	17	3
KBr (*n* = 3)	0	0	0	1	0	0	0	0	0	0
LEV (*n* = 1)	0	1	0	0	0	0	0	0	1	0
PB + KBr (*n* = 66)	15	11	18	24	11	4	8	1	12	1
PB + LEV (*n* = 5)	1	2	5	3	0	0	0	0	1	0
PB + KBr + LEV (*n* = 10)	4	4	4	4	4	3	3	0	4	0
Other AED (*n* = 7)	1	1	1	3	2	0	0	1	2	0
Count	36	38	44	50	21	10	16	2	37	4
Percentage of all reviewed dogs (*n* = 185) in %	19.5	20.5	23.8	27.0	11.4	5.4	8.6	1.1	20.0	2.2

### Prospective Evaluation of Cutaneous Signs

Within this study part, 137 dogs (74.5% idiopathic epilepsy and 25.5% structural epilepsy) fulfilled the inclusion criteria. Of the 137 dogs, 10.9% (95% CI 6.8–17.7%, 15 dogs) presented with dermatologic signs of varying extent, which appeared after initiation of AED therapy. All of the dogs with dermatologic signs were diagnosed with idiopathic epilepsy except for one (P5) with ambiguous results in neurologic examination, which were not consistent with idiopathic epilepsy. Detailed owner interrogation revealed that three of the dogs already had mild dermatologic signs before onset of seizures which worsened after AED initiation (Table 5). All dogs were treated with PB. Twelve dogs received PB monotherapy, and three were treated with a PB combination therapy (PB/LEV, PB/KBr, PB/LEV/KBr). One of the dogs (P14) developed dermatologic signs shortly after initiation of LEV therapy, the others within days to several months after initiation of PB.

**Table 5:** **Prospectively collected cases of skin lesions under antiepileptic treatment**.

No.	Signalment	Antiepileptic therapy	Adverse events	Dermatologic signs prior to AED therapy	Description of dermatologic signs after initiation of AED	Chronological appearance of dermatologic signs	Discontinuation of AED therapy	Classification of skin lesions according to Naranjo et al.	Patch test	Intra-dermal skin test
P 1	Mixed-breed, male, 8a	PB	Dermatologic signs	None	Severe erosive perianal inflammation with purulent secretion	Several months after initiation of AED	Yes, but only for 2 weeks, no improving of skin lesions during that period	Possible	Positive (2+)	Negative
P 2	Saluki, male-neutered, 5a	PB	Dermatologic signs	None	Erosive-crusty dermatitis, generalized spread, especially in the face, at the testicles and oral mucosa	Several weeks after initiation of AED	Yes → complete remission within 1–2 weeks	Probable	Negative	Negative
P 3	Mixed-breed, male, 4a	PB later combination therapy PB/LEV	Dermatologic signs	None	Pruritus, crusty lesions at the hind limbs and rhinarium	Weeks to months after PB initiation	No	Possible	Negative	Positive (+ +)
P 4	Golden Retriever, female-neutered, 7a	PB	Dermatologic signs	None	Pruritus and alopecia at the limbs	Several months after initiation of AED	No	Possible	Negative	Negative
P 5	Mixed-breed, male, 3a	PB	Dermatologic signs	None	Erosive and crusty lesions perioccular, at the limbs and foot pads	Several days after initiation of AED	Immediately after appearance of dermatologic signs → complete remission within 1–2 weeks	Probable	No definite result (± )	Negative
P 6	Mixed-breed, male, 5a	Combination therapy PB/KBr	Dermatologic signs	None	Pruritus and alopecia at the limbs, crusty lesions at the ears and the inguinal region	Several months after initiation of AED	No	Possible	Negative	Negative
P 7	Mixed-breed, male-neutered, 8a	PB	Dermatologic signs, changes in behavior	None	Pruritus and crusty lesions around the eyes, at the ears, at the armpit area and breast	Several weeks after initiation of AED	Yes, for several weeks, improving of skin lesions during that period	Probable	–	–
P 8	Mixed-breed, male-neutered, 2a	PB	Dermatologic signs, ataxia	None	Erosive and crusty lesions in the face and at the limbs	Several weeks after initiation of AED	No	Probable	–	–
P 9	Hovawart, male-neutered, 2a	PB	Dermatologic signs	None	Erosive lesions and discoloration of oral mucosa	Weeks to months after drug initiation	No	Possible	–	–
P 10	Australian shepherd, male-neutered, 8a	PB	Dermatologic signs	None	Generalized cornification of the skin, generalized spread papules and nodes	Several months after initiation of AED	No	Possible	–	–
P 11	Yorkshire terrier, male, 7a	PB	Dermatologic signs	Moderate pruritus because of flea several months before	Severe pruritus and skin rash (pustules, papules, macule) in the armpit region and at the flanks	4 days after initiation of AED	Yes → complete remission within 1 week, never received PB again	Probable	–	–
P 12	French bulldog, female-neutered, 7a	PB	Dermatologic signs: PU/PD, polyphagia	Mild allergic skin disease (pruritus) already before AED initiation	Aggravation of pruritus and appearance of crusty lesions at the rhinarium and hypotrichous areas and erythema at the limbs, perianal inflammation	Weeks to months after drug initiation	No	Doubtful	–	–
P 13	Australian shepherd, male, 3a	PB	Dermatologic signs	None	Generalized spread, severe erosive, necrolytic dermatitis, especially at the mucocutaneous boundary, foot pads, testicles, perianal, and in the inguinal and armpit area	1–2 weeks after initiation of phenobarbital therapy	Immediately after appearance of dermatologic signs – >complete remission within 1–2 weeks	Probable	–	–
P 14	American bulldog, male, 2a	PB/KBr	Dermatologic signs, especially under levetiracetam	Mild pruritus already before AED therapy	Severe pruritus	Days after initiation of LEV therapy	Immediately after appearance of dermatologic signs → complete remission within several days	Possible	–	–
		LEV (for several days)								
		Later additionally imepitoin								
P 15	Labrador retriever, male-neutered, 9a	PB	Dermatologic signs, polyphagia	None	Generalized spread, crusty, erosive lesions, alopecic areas, hypotrichosis at the limbs	Weeks after drug initiation	No, but improvement of signs under corticosteroids	Possible	–	–

**Figure 1:**
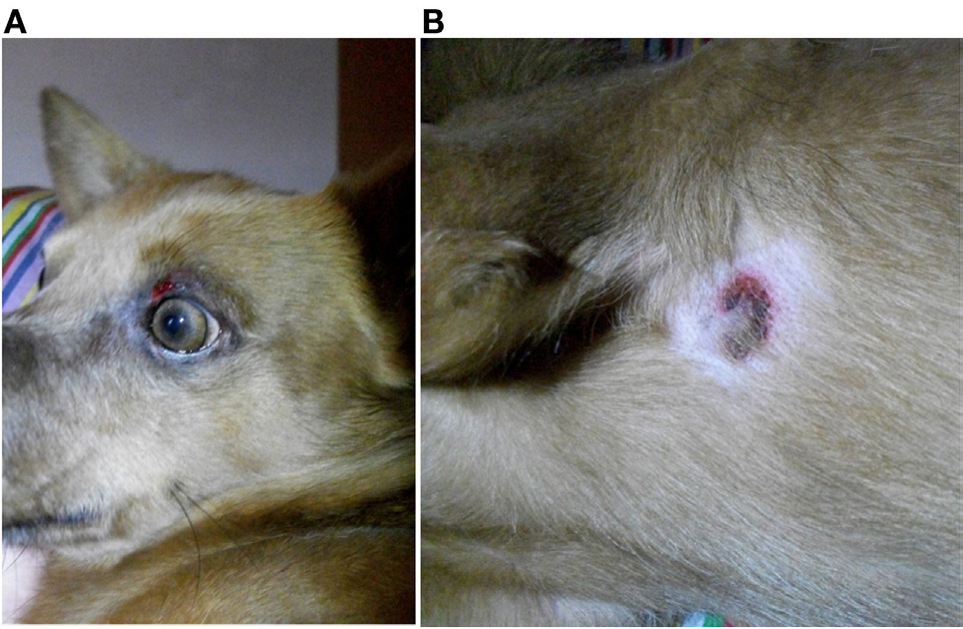
**Dog P7 (mixed-breed, male-neutered, 8 years) developed a generalized, erosive-crusty dermatitis, especially in the face (A) and in the axiallry region (B) several weeks after initiation of PB; clear improvement of dermatologic signs after PB withdrawal**.

**Figure 2:**
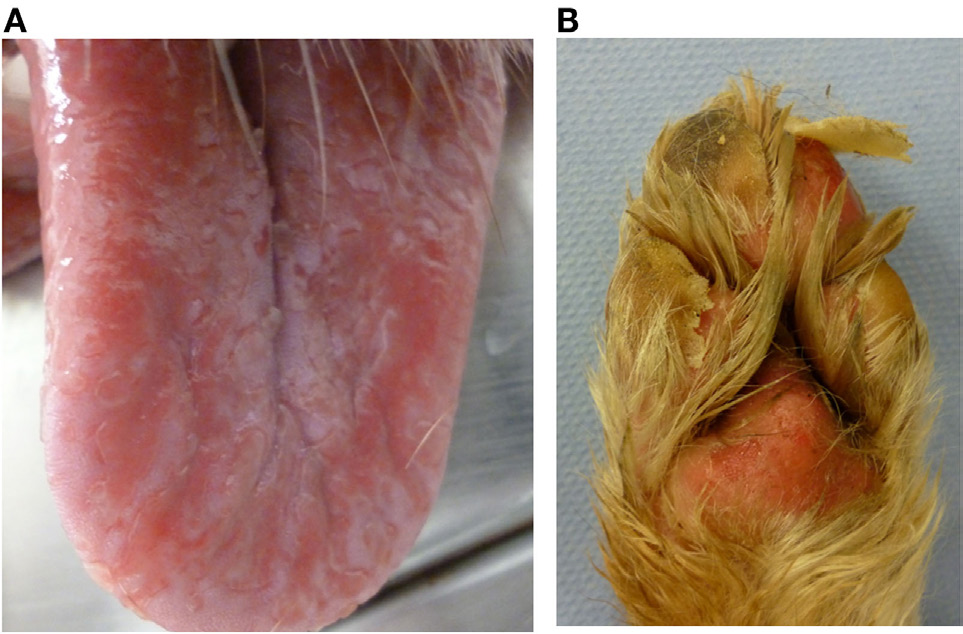
**Dog P13 (Australian shepherd, male, 3 years) developed a generalized, severe, erosive necrolytic dermatitis, especially at the oral mucosa (A), at the mucocutaneous junctions and the foot pads (B) 1–2 weeks after PB initiation; additionally affected were the testicles and the perianal, inguinal and axillary areas**. After withdrawal of PB therapy, the skin lesions resolved completely within 2 weeks.

Nine dogs (P1, P3–6, P11–13, and P15) underwent complete dermatologic examination performed by a diplomate or resident ECVD, which included detailed dermatologic history and visual inspection of the lesions in all examined dogs as well as cytologic examination (*n* = 8), skin scrapings (*n* = 4), and bacteriological culture (*n* = 2). The remaining six dogs were examined by other clinicians, lacking further dermatologic tests. The cutaneous signs were described as severe with extensive erosions or epidermal necrosis leading to skin detachment in four dogs (P2, P5, P7, and P13). Eight dogs showed moderate signs, most notably papules, pustules, erythema, and crusty lesions, and three dogs showed mainly pruritus and/or alopecia. Particularly affected were the face, especially the periocular region, the mucocutaneous boundaries, the inguinal region, and the limbs (Table 5). Skin scrapings revealed *Sarcoptes* mites in dog P15. In this dog, antiparasitic therapy led to partial improvement of cutaneous signs. In the other 14 dogs, no other cause for the cutaneous lesions was identified. Skin biopsy was performed in one dog (P13) and indicated presence of erythema multiforme. AED therapy was withdrawn in seven cases (six dogs with PB and one dog with LEV). Cutaneous signs dissolved completely in five dogs within approximately 2 weeks (P2, P5, P11, P13, and P14). In one dog, a clear improvement, although no complete remission, was noticeable (P7), and in another dog (P1), appearance of severe seizures despite concomitant loading with KBr led to reinstitution of PB therapy after 2 weeks. Treatment for the cutaneous lesions included anti-inflammatory and/or antimicrobial topicals (*n* = 8), antiparasitic agents (*n* = 6), and short-term systemic prednisolone (*n* = 3). Treatment led to improvement of signs in all of the cases. However, this improvement was not complete and only temporary while topical or systemic anti-inflammatory medication was given. Based on the individual patient’s history, the examinations and the information obtained by standardized questionnaires, the Naranjo index was applied to classify the cutaneous signs as probably due to AED therapy in 40.0% (six dogs, all treated with PB monotherapy), and as possible (eight dogs; 53.3%) or doubtful (one dog; 6.7%) in the remainder (Table 5). As an example, the skin lesions of dog P7 and P13 are depicted in Figures 1A,B and 2A,B. Both dogs had developed dermatologic signs several weeks after initiation of PB therapy and were both classified as probably drug-induced ADR.

### Evaluation of Patch Test and Intradermal Test

Six dogs with cutaneous signs occurring under antiepileptic therapy (four PB monotherapy, one PB/LEV, and one PB/KBr) were tested with PT and IDT. Cutaneous signs were classified as probable due to AED therapy in two of these dogs, and as possible due to AEDs in the other four dogs. The clinical details of these patients are listed in Table 5 (P1–P6) and are described below. All six dogs underwent complete dermatologic examination: ectoparasites were not found in any of these dogs but other possible causes for dermatologic signs such as food or environmentally induced atopic dermatitis could not be precluded assuredly. This fact has been regarded in ranking the probability of an adverse event using the Naranjo index.

**Figure 3:**
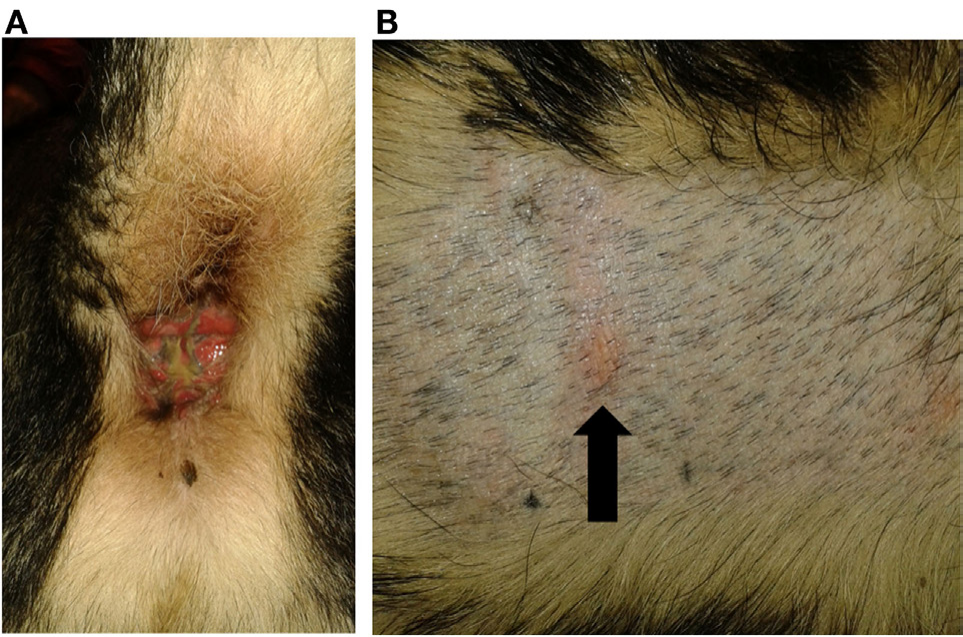
**Dog P1 (mixed-breed, male, 8 years) developed a severe erosive perianal inflammation with purulent secretion after PB initiation (A)**. Patch test revealed a positive reaction to the 10% phenobarbital (arrowed) **(B)**.

One of the tested dogs (P1; possible ADR) showed a strong positive reaction in PT to the 10% petrolatum-solved PB (Figures 3A,B). The other patch test sites (negative control and 5% dilution) as well as intradermal reactions were negative. This dog (P1, 8a) had been treated with PB for several years and developed a severe erosive perianal inflammation a few months after initiation of medical treatment with PB. After the positive PT, the AED therapy was changed to potassium bromide by first starting a loading dose of KBr (600 mg/kg per day, over 4 days) and subsequent gradual decrease of PB over a period of 2 weeks. Approximately 2 weeks after cessation of PB, a period of cluster seizures re-occurred, thus PB therapy was resumed. During the short period off PB, the perianal inflammation did not improve noticeably. It should be noted that this dog already had regular cluster seizures while being treated with PB.

**Figure 4:**
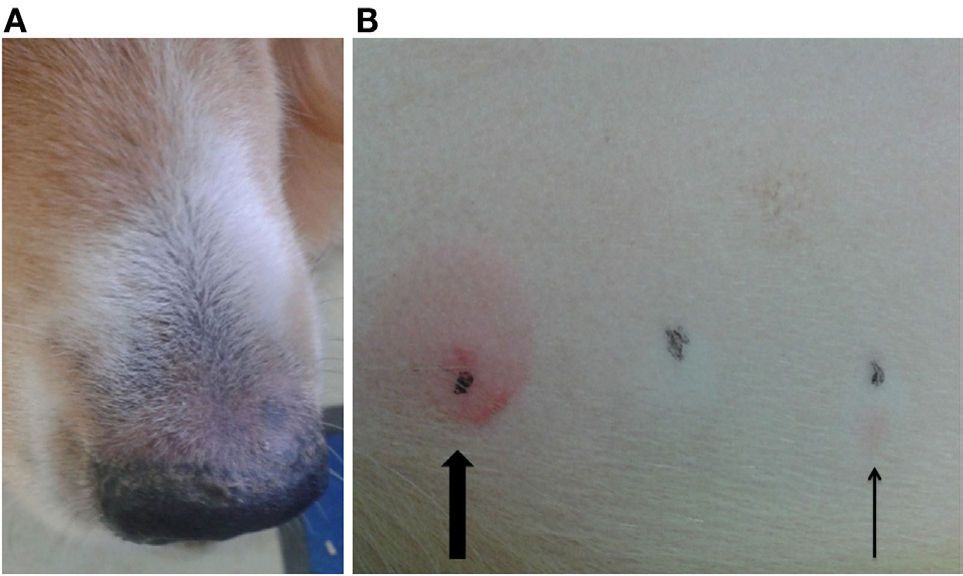
**Dog P3 (mixed-breed, male, 4 years) developed pruritus and crusty lesions at the hind limbs and the rhinarium (A)**. Intradermal test revealed a positive reaction at the phenobarbital injection site (slim arrow), compared to positive control (big arrow) **(B)**.

Another dog (P3, possible ADR) was positive on IDT for PB, but negative on the patch test (Figures 4A,B). This dog was treated with PB for 3 years and showed pruritus and crusty lesions at the hind limbs and rhinarium. The cutaneous signs appeared shortly after PB therapy was started. PB therapy was not discontinued in this dog due to the severe cluster seizures seen in that patient.

A third dog (P5) had an ambiguous patch test result against the 10% PB. This dog received PB only for 1 week. Therapy was then discontinued because of the sudden appearance of severe pruritus and alopecia at the limbs, crusty lesions periocular, at the ears and the inguinal region and erosive lesions with ulcera at the foot pads. After cessation of PB treatment, the skin lesions resolved completely within 2 weeks. This history supported a cutaneous adverse reaction to PB.

The other three tested dogs (P2, P4, and P6) with skin lesions developed under PB treatment were negative on IDT and PT.

None of the tested healthy laboratory beagle dogs showed a positive reaction on PT or IDT, neither did any of the private-owned dogs of the negative control group (epileptic dogs under PB treatment without any registered cutaneous signs – currently or previously to AED therapy).

Sensitivity, specificity, and positive and negative predictive value of PT and IDT are listed in Table 6.

**Table 6:** **Sensitivity, specificity, positive and negative predictive value of patch testing and intradermal testing for AED hypersensitivity**.

	Sensitivity (%)	Specificity (%)	Positive predictive value (%)	Negative predictive value (%)
Patch test	16.7	100.0	100.0	84.4
Intradermal skin test	16.7	100.0	100.0	84.4

## Discussion

This study was initiated based on our own unpublished clinical observations, which suggested simultaneous appearance of skin disease and epilepsy more commonly than previously reported. This prompted further investigations of the prevalence of cutaneous signs and their relationship to AED treatment. Subsequently, we confirmed a surprisingly high prevalence of cutaneous signs with 20% in the retrospective data analysis, and identified 15 dogs with potentially AED-caused cutaneous signs in the prospective study part. The prevalence in the prospectively evaluated group was 10.9% (15/137) and exceeded the prevalence of hepatopathy and pancreatitis in this investigation.

Dermatologic examination in our dogs was unable to delineate other etiologies than drug hypersensitivity, such as ectoparasite manifestations or allergies in all but one dog (P15), but not all diagnostic tests were applied to each dog (31). Literature describes a variety of cutaneous signs caused by an ADR, ranging from urticaria and angioedema (immediate drug hypersensitivity), pruritus, or exanthema to severe generalized syndromes such as lupoid/pemphigoid reactions or SND (15). March et al. retrospectively evaluated the appearance of SND/MEN with chronic PB administration in dogs (9). The two main differences between the results of this study and our results were the duration of AED treatment before onset of skin lesions (a median of 7 years in March’s study and weeks to months in our cases) and the severity of skin lesions (more severe and potentially life-threatening SND/MEN in March’s study versus predominantly mild to moderate cutaneous signs in our study). March et al. focused on a PB-induced hepatopathy, while we evaluated any reaction to the drug which may be the explanation for this difference.

In order to further highlight the relationship between AED treatment and appearance of cutaneous signs the ADR probability scale of Naranjo et al. (26), a classification system validated in human medicine (32, 33), was applied. This scale estimates the likelihood of an ADR based on a number of criteria (Table 1). It is particularly helpful when proof of a drug reaction cannot be achieved under clinical circumstances with a drug challenge for ethical reasons, and also accounts for missing information on particular details, e.g., whether cutaneous signs were present prior to AED treatment. The individual patient history was reviewed with regard to the chronological relationship between initiation of drug treatment and appearance of dermatologic signs, the precise localization and appearance of skin lesions and whether signs improved after withdrawal of AED whenever feasible. Based on this evaluation, 2 of the 37 retrospective cases and 6 of the 15 prospective cases were classified as probably suffering from anticonvulsant drug hypersensitivity.

Another explanation for the high prevalence of dermatologic signs in epileptic dogs may be that there are common immunologic mechanisms underlying both the skin disease and the epilepsy. Several different facts support this hypothesis: food allergies or asthma might increase the risk of developing seizure disorders in humans and animals (34, 35). Mouse models show inflammatory pathways involved in allergic diseases that may also be activated in the brain and may cause epilepsy (36). Dietary supplementation with either ω3 fatty acids or medium chain triglycerides are used to improve seizure control in individuals with refractory epilepsy (37–41) and are also successfully administered to dogs with atopic dermatitis (42, 43). Thus, there may be a link between allergic diseases and epilepsy leading to cutaneous signs in epileptic dogs independent of antiepileptic therapy. Investigations indicate that in humans, genetic factors predispose for developing an adverse reaction against AED (23). In contrast, in this study no breed predisposition for developing adverse events could be determined.

Secondary goal of our study was to evaluate PT and IDT as potential diagnostic methods in suspected ADRs against AEDs in dogs. In human, medicine PT is already frequently used to confirm AHS (44). In previous studies, the positive predictive value was much higher than the negative predictive value and the validity of the PT varied between the different AEDs (highest for carbamazepine, lowest for PB) (28). In contrast, in veterinary medicine, there are only few reports about the use of skin tests as diagnostic tools for ADRs. In 2008, Murayama described PT to identify an allergic reaction to ingredients of a shampoo in a miniature schnauzer (20, 45). Apart from this, PT proved useful as an aid to choose the ingredients of an elimination diet in dogs with adverse food reaction (27), and IDT is widely used to identify offending environmental allergens in dogs with atopic dermatitis (30). Those established methods were used as guidance to develop the skin tests in our study. Both tests showed a high negative predictive value, none of the healthy controls and none of the epileptic dogs under PB therapy without dermatologic signs showed a positive reaction in either PT or IDT. In the positive control group (*n* = 6), one of the tested dogs was positive on patch test, and one of the dogs showed a positive reaction on IDT. The contradictory results of PT and IDT in these two dogs might be due to different underlying immune mechanisms. An IDT evaluated shortly after injection as in this study is more sensitive for immediate hypersensitivity reactions, in contrast PT is more sensitive for delayed-type hypersensitivity reactions (46–48). A late judged IDT (evaluation of skin reaction after hours or days) might have revealed other results but was not feasible in this study. Thus, PT and IDT in the present form were insufficient to confirm suspected AED hypersensitivity, which might indicate that other mechanisms than drug hypersensitivity were involved, or that a modification of the skin test protocol with higher drug concentrations or different application methods is needed.

This study also provided insights into occurrence of adverse events of AEDs previously reported (1, 2, 49) in comparison to the appearance of dermatologic signs. Ataxia and sedation occurred in approximately a quarter of the patients. These are known side effects of AEDs (2, 3), occur most common during the first weeks of therapy and tend to improve (3). Hepatopathy, pancreatitis, and blood dyscrasias are of major concern in literature (5, 6, 50–52), but occurred less frequently than dermatologic signs in this study population (hepatopathy in 1/50, pancreatitis in 1/20, neutropenia in 1/30). This should be considered when educating clients about side effects of AED therapy.

Limitations of the study are certainly that we did not use other approaches to confirm AED hypersensitivity. A major limitation of this study is that not all of the dogs were examined by a veterinary dermatologist and that further dermatologic examinations were not performed on each dog. Although cutaneous signs in a proportion of dogs were also suggestive of MEN, histopathologic confirmation was only obtained in one dog and sonography of the liver to show typical sonographic changes of MEN was not performed because of lacking owner-acquiescence. Also none of the dogs, in whom skin reactions disappeared after cessation of anticonvulsants, underwent a second challenge with the drug for ethical reasons, although this would have confirmed AED hypersensitivity reaction more reliably.

In summary, based on the results of this study, dermatologic signs appear realtively frequently in epileptic dogs treated with AED. The pathomechanism of those cutaneous signs needs to be further investigated. Moreover, patch testing and intradermal testing should be evaluated for the diagnosis of a suspected drug hypersensitivity in a larger number of dogs on AED with cutaneous signs. Changes to the protocol of those tests may increase the positive predictability while maintaining the safety of those tests.

## Author Contributions

TK: designed the experiments, conducted the experiments, and wrote first draft of the paper. RM: designed the experiments and provided corrections and feedback on first drafts of paper. BD: assisted in the experimental part on research dogs. AF: designed the experiments, and provided detailed corrections and feedback on interpretations of results and first drafts of paper.

## Conflict of Interest Statement

The authors declare that the research was conducted in the absence of any commercial or financial relationships that could be construed as a potential conflict of interest.
